# Water Sorption by Different Types of Filter Media Used for Particulate Matter Collection Under Varying Temperature and Humidity Conditions

**DOI:** 10.3390/ijerph17145180

**Published:** 2020-07-17

**Authors:** Kamila Widziewicz-Rzońca, Malwina Tytła

**Affiliations:** Institute of Environmental Engineering, Polish Academy of Sciences, 34 M. Skłodowska-Curie St., 41-819 Zabrze, Poland; malwina.tytla@ipis.zabrze.pl

**Keywords:** particulate matter, gravimetric, filters, PTFE, nylon, glass, quartz, monitoring campaigns, water contents, Karl Fischer titration

## Abstract

The present study describes the effects of temperature and humidity on the level of water absorption by filter blanks most popularly used for gravimetric analyzes of particulate matter (PM) and the effects of those on the accuracy of its weight measurements. The main parts of the research quantified the effect of temperature and humidity conditions on water contents quartz fiber (Q), fiberglass (G), PTFE, and nylon (N) filters. Supplementary studies were conducted to estimate the effects of temperature, humidity and material on mass loss/gain and the shape of water retention. All chemical analyses of water contents were performed by the Karl Fischer titration method. The results indicate that quartz filters are the most susceptible to the variations in water contents under changing humidity levels and therefore, less suitable to high accuracy determinations of PM mass compared to nylon or glass filters; PTFE performed the best due to their hydrophobicity. For PM water contents determinations, the best choice of filter media is PTFE. Although many other factors determine the choice of filter type for PM analyses, the present study is an important contribution to knowledge of assessing the suitability of different types of filter material for specific measurements.

## 1. Introduction

The World Health Organization (WHO) considers clean air a fundamental human need and defines this as “a basic requirement of human health and well-being” [1,2]. One of the most important pollutants regarding human health is particulate matter (PM). A standard method for its determination is based on sampling and weighing of the PM on filters and is defined by legislation (Council of the European Union, [3]). Recently, public awareness has moved toward PM_2.5_ and PM_1_ particles, mainly due to the health effects posed by those fractions: mortality, respiratory and cardiovascular morbidity, and inflammatory airway response including asthma [4,5]. The Total Suspended Particles (TSP) and PM_10_ mass measurements are well standardized; but there are no recommendations for the instruments or techniques used for the investigation of ultrafine particles. Since ultrafine particles reach high concentrations but their mass is often very small, measurements of particles in ultrafine or even sub-micrometer ranges are more commonly based on particle number rather than mass concentration. Despite this, efforts to understand health impacts of fine and ultrafine particles means that the actual mass of PM available for the measurement process becomes smaller, which in turn makes the PM mass measurement accuracy more and more important. 

Generally, the two methods for measuring PM mass are direct gravimetric mass measurement and mass approximation based on known chemical composition [6,7]. Discrepancies between measured and reconstructed PM mass is due to the measurement process, including: (1) vaporization of some organic compounds adsorbed on filters, (2) volatilization of ammonium nitrate (3), estimation of chemical species not directly measured, (4) transport losses especially in case of quartz and glass fiber filters, (5) volatilization of some species due to elevated temperature in the sampling inlet, (6) disruptions in the performance of PM samplers, and (7) retention of water vapor [8,9,10]. The amount of retained water is the sum of PM-bound water, and the amount of water absorbed by filter media [11]. Quantitative determination of the filter-bound (blank) water contents is crucial for the proper assessment of the actual mass of PM. The mass of PM filters without any collected PM varies from filter to filter depending on air temperature and humidity [12]. While the changes between the mass of sampled and unsampled PM filters are controlled by conditioning requirements, the contribution of water increases measurement uncertainty between different types of unsampled (blank) filters; how this is affected by changing temperature and humidity conditions is poorly recognized. The maximum effect of air for unsampled filters is usually quantified by the constraints on mass change during multiple weighing sessions [8,13,14]. Currently, few papers recognize the amount of blank filter water contents by using thermogravimetric (TG) analysis [15] or the Karl Fischer titration method [9,16]. The water content of unsampled filters depends not only on conditioning and “history” of filter, i.e., ambient air conditions during filter packaging or transport. The amount of water absorbed or evaporated from the surface of the coated (sampled) filter is due to the behavior of filter and the sample which are physically and chemically completely different. Previous studies quantitatively describe the influence of air humidity and temperature on sampled filters [16]. Water content depends on whether the relative humidity is above or below the deliquescence or efflorescence relative humidity of the PM compound, respectively [16,17,18]. For PM chemical compounds, these points lie between 75% RH and 35% RH [18]. Water retention is a problem during long measurement campaigns. Changes in the ambient air humidity can cause changes to the mass of PM collected over days or weeks. The removal of PM-bound water could be done by filter heating; however, after treatment, some volatile compounds could be partially lost. Preliminary heating or dehumidification of blank filters under temperature higher than ambient air conditions followed by an equilibration step does not guarantee that the water contents will be restored to the starting conditions due to significant hysteresis in the water adsorption-desorption pathways [13]. Water vapor is poorly controlled, even in laboratory conditions. The variation of temperature and humidity in the weighing room can modulate filter-bound water contents and the hydrophilic or hydrophobic nature of the filter medium, its relative thickness, pore size and specific surface area, to name a few properties.

There are many filter types used in ambient air sampling [6]. Filters used for PM collection are characterized by different physical and chemical features, and none of them are universal enough for all analyses. Each filter type poses some advantages for either the collection or analysis of a set of some compounds. The most decisive criteria in selecting filters for measurements are: collection efficiency (filters should remove 99% of suspended particles), mechanical and chemical stability, temperature stability, blank concentrations, flow resistance, loading capacity, and their cost and market availability [7]. For example, Teflon filters are efficient for the collection of PM due to lower susceptibility to mass lost during crimp-sealing within the filter holder; they typically have low blanks and very high initial particle capture efficiency; they also maintain integrity even with the most aggressive total digestion procedures for trace elements [19]. Quartz filters are typically used for organic carbon/elemental carbon determination because the analytical methods used (e.g., thermal/optical reflectance and transmittance) involve high-temperature thermal desorption. Nylon filters have very high collection efficiency (>99% for 0.01 µm particles) and are excellent for X-Ray analysis and for HNO_3_ collection [20]. The most suitable filter material for water determinations should be hydrophobic. The simple gravimetric analysis of water contents is estimated based on difference between the mass of equilibrated and non-equilibrated filter is not sensitive enough to reflect the actual amount of water retained in the conditioning step. Thermogravimetric methods cannot quantify the very low amount of water usually found in filter blanks. The method that has high sensitivity for low water contents is the Coulometric Karl Fischer titration, which is able to detect amounts as small as 10 μg of absolute water; and therefore, it is preferred for the highest precision needs [21]. The knowledge regarding concentration of volatile chemicals such as ammonium nitrate, certain organic compounds as well as liquid water which can vary during sampling, transport storage or sample analysis is of much importance from the point of view of compliance with Air Quality Standards. For example, liquid water can be removed by lowering the relative humidity, surrounding the sample, by laboratory equilibration, by heating the sampled air stream or even by selectively denuding the airstream of water vapor. Recommendations regarding conditions of sampling and filters equilibration are of much importance for compliance monitoring. The view taken in this study is that a standard method for the determination PM concentration must focus primarily on making the measurement of filter blanks mass and PM mass as reproducible and accurate as possible.

The motivation of this study was to quantify the influence of small variations of temperature and humidity in the ranges close to the recommended equilibration conditions on the gain/loss of filter media mass after 24 and 48 h of conditioning time; this influence was calculated as ΔM (mean mass ±SD) µg. The second aim was to measure the water contents in filter media subjected to the exposure to the variations in temperature (18 °C; 22 °C) and humidity (20; 30; 40; 50; 60; 70, 80; 90% RH).

A short study was also undertaken to assess to which extent the type of filter material modulates connections between filter mass and its water contents (Figure S3). An investigation was made to found positive or negative correlations (R^2^) between average filter mass (m) and measured water content (w) under different moisture and temperature conditions.

## 2. Materials and Methods

### 2.1. Experimental

**Figure 1:**
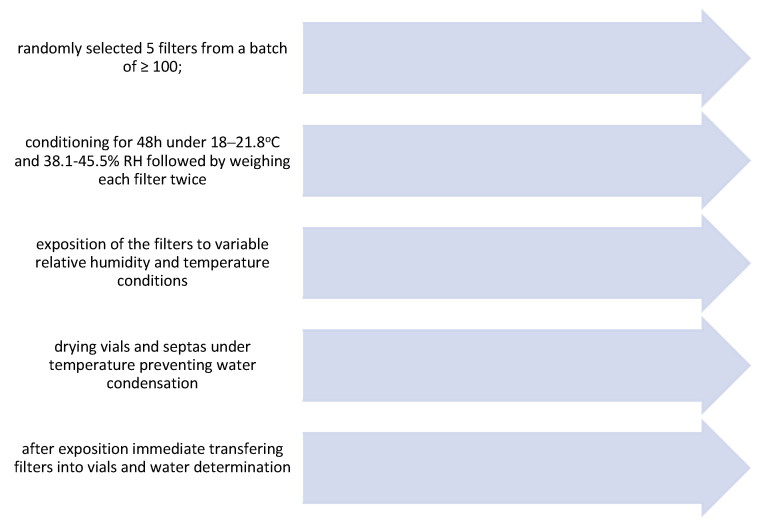
Methodological scheme used for assessing the influence of temperature and humidity variations on the water content of filter blanks.

The experimental part of the study lasted a few weeks and included conditioning for 48 h, filter exposure for 24 h and water determination on the following day in consecutive cycles (Figure 1). Four types of filters were tested quartz fiber filters (Q), fiberglass (G), PTFE filters (PTFE: 2 µm, polypropylene supported for PM2.5 and PTFE plane, polypropylene baked 0.5 µm) and nylon filters (N) (all produced by Whatman, GE Healthcare Bio-Sciences Corp; Piscataway, NJ, USA). Humidity conditions used in the experiments were 10, 20, 30, 40, 50, 60, 70, 80, 90% RH under 18 °C and 22 °C separately. PTFE filters were treated as one group since its behavior and response to changing temperature/humidity conditions were similar. Sixteen independent experiments were performed in duplicate (eight humidity ranges, two temperature ranges, four types of filters, and five replicates of each filter), resulting in a total of 10,240 filters being tested.

Filter mass was assessed using a balance (range: 0.001–0.1 mg; resolution 2 µg; Mettler Toledo, Columbus, Ohio, USA). Prior to weighing, filters were equilibrated at a constant temperature. Conditions in the weighing room were maintained at 18–21.8 °C and 38.1–45.5% RH and controlled with a wireless temperature and humidity monitoring system. Slightly different equilibration conditions were used compared to those recommended by the EN 12341:2014 [3] standard (19–21 °C and 45–50% RH values). Rather low range of conditioning temperature used in this study was chosen based on the results presented by [12], which generally (in case of Teflon filters) allows a good mass stability. A wider range of conditioning temperature cannot be set due to the balance requirements, which was housed in conditioning room in order to reduce temperature fluctuations inside the balance. After the first weight measurement, the filters were returned to Petri slides, and equilibrated for another 24 h in the weighing environment, according to the microbalance operating manual. The filter mass was calculated as follows:

M = M_24_ + M_48_/2 (1)

where M is the filter mass, calculated as mass averaged between 24 h and 48 h of equilibration.

If the difference in filter mass between the 24 h and 48 h weighing was greater than 40 µg, the filter was rejected from analysis according to EN 12341:2014 [3] and EN 12341:1999 [22] recommendations, due to significantly departing mass values. 

The equilibrated filters were subjected to various temperature and humidity conditions (18 °C and 22 °C under 20; 30; 40; 50; 60; 70; 80; 90% RH) in a climatic chamber (Vitromat, Mytron Bio-und Solartechnik GmbH, Heiligenstadt, Deutschland) connected to a Ventage Pro 2 weather station (Davis Instruments, Hayward, CA, USA); two independent temperature and humidity sensors were integrated in the climatic chamber for a more accurate detection circuit, which is critical for quality control purposes. The exposure time was set to 24 h. Filters inside the chamber were placed into individually labeled and opened Petri-slides. After exposure, the filters were immediately moved into dried vials (250 °C, 24 h; stored in a desiccator) and closed by aluminum septum caps (this operation was performed inside the AtmosBag—Sigma Aldrich (St. Louis, MO, USA)—to eliminate sorption of additional portions of atmospheric water). Filters were then subjected to water determinations in a Karl Fischer analyzer including 831 KF Coulometer (Metrohm AG, Herisau, Switzerland) equipped with an oven (874 Oven Sample Processor; Metrohm). Karl Fischer coulometric titration is a classic titration method in chemical analysis to determine trace amounts of water in a sample based on the Bunsen reaction. In the coulometric version of this titration method, the endpoint is determined electrochemically. In other words, water present in a filter sample is consumed by the KF reaction until a slight excess of iodine has accumulated, which is detected by double Pt electrodes and defines the end of the titration. Due to temperature limitations related to individual melting points of each filter type, the maximum temperature of water release was adjusted to achieve complete drying while keeping the temperature below filter’s melting points—quartz stable above 1600 °C. An excessive filter heating can cause partial volatilization of the filter material or its decomposition. When adjusting extraction temperatures, we followed the operating temperatures given in the product characteristic card—for example, nylon filters have an operating temperature <135 °C. Particulate Matter (PM_2.5_) Speciation Guidance Document [2] provides melting points for filtration media, which are generally lower than the characteristic cards (60 °C for nylon and PTFE filters). However, lowering the extraction temperature causes a significant elongation of analysis. The water extraction temperature was set to 220 °C for quartz and glass filters and 60 °C for nylon and PTFE.

Moisture determination began when the vial was moved into the oven, water was being vaporized and channeled into measurement cell by a carrier gas, where it was finally measured. The detection of the endpoint included the amount of water resulting from its presence in the measurement system and the carrier gas. This value was automatically removed from the analysis in the conditioning step. Ambient air with an airflow of 50 mL/min was used for transferring samples into titration cells. Before this step, the air was filtered and dried by using molecular sieves (0.3 nm pore size regenerated under 300 °C). The needle for gas inlet was washed in methanol (HPLC grade, Baker) each day to prevent the tubing from clogging up. Different volatile substances are systematically desorbed from filters during the heating step; therefore, it is crucial to clean the system routinely. As a titration solvent, the Karl Fisher reagent (150 mL, Hydranal-Coulomat AG-Oven, Fluka, Buchs, Switzerland) was used. The parameters of the method were that the minimum value of the voltage difference between the indicator platinum electrodes was set at 50 mV, the relative stop drift value was 5 μg/min and the total duration of the analysis (extraction time) was 3000 s. The baseline drift–blank water contents were estimated by measuring water contents enclosed in empty vials (6 mL v/v) in conditions corresponding to those in the climate chamber during the filter exposure. Water desorbed from the blank sample was subtracted from the filter-bound water amount, which gave the final result. Both the operative blank samples and tested samples were measured in five replicates. Direct measurement of the contribution of blank water amounts, the humidity of the air inside the vial, the adsorption on the vial walls, and the content of the plastic vial cap were assessed by comparing the blank signal with the sample signal on a single graph (overlay curves). The time interval for downloading data from the Davis system (humidity and temperature conditions) was set to 1 h. Therefore, 24 values for each parameter were obtained from one measurement cycle and the temperature/humidity conditions are presented as values averaged over 24 h. The filters were handled with great care during all operations including weighing, exposure, and finally chemical analysis. 

### 2.2. Method Validation

To evaluate the performance of the developed method, water was determined in two certified reference materials: Hydranal Water Standard KF-Oven 220–230 °C (HYD; Fluka Analytical) and Apura Water Standard Oven 1% (WSO; ACS Merck KGaA, Darmstadt, Germany), containing 5.55 ± 0.05 and l.0 ± 0.03% of water, respectively. According to information prepared by Fluka (Hydranal Manual) and given in certificate of analysis of both CRMs, the optimum sample quantity was 80 mg for WSO and 200 mg for HYD (3 replicates). 

The limit of detection (LOD) and quantification (LOQ) were calculated by measuring an analyte (water) concentration corresponding to the sample blank value plus three standard deviations and ten standard deviations, respectively, as shown in the following equations:

LOD = X_b1_ + 3S_b1_ (2)

LOQ = X_b1_ + 10S_b1_ (3)

where X_b1_ is the mean concentration of the blank and S_b1_ is the standard deviation of the blank (10 replicates).

For blanks prepared under laboratory conditions (dried, empty vials capped in the room, where the KF apparatus was kept), a typical intra-day variability in the blank water contents was LOD = 42.21 ± 13.3 μg and LOQ = 42.21 ± 44.35 μg. For blanks (dried, empty vials kept in the desiccator and capped in the ATMOS bag) LOD and LOQ values were lower (LOD = 20.6 ± 18.73 μg; LOQ = 20.6 ± 62.4 μg).

### 2.3. Statistical Analyses

All statistical analyses were performed using Excel (MS Office, Redmond, WA, USA) and Statistica 13 Package (StatSoft, Cracow, Poland). The average filter mass was calculated as the arithmetic mean from two subsequent filter’s weighing (after 24 and 48 h). The difference in filter weight (a total of five filters of each type) between the first and second weighing was calculated as the standard deviation (SD) value from the averaged filter mass. Differences in the total filter mass between all weighing sessions within the batch of filter of one type was calculated as standard deviation (SD) of repeatability (µg). To check whether the average mass of filter blanks was positively correlated with the change in humidity/temperature, a one-way ANOVA was performed (*p* < 0.05).

## 3. Results

### 3.1. The Effect of Temperature and RH Variations in the Range Recommended by EN 14907:2005 (E) [23] on the Mass of Filter Blanks

**Figure 2:**
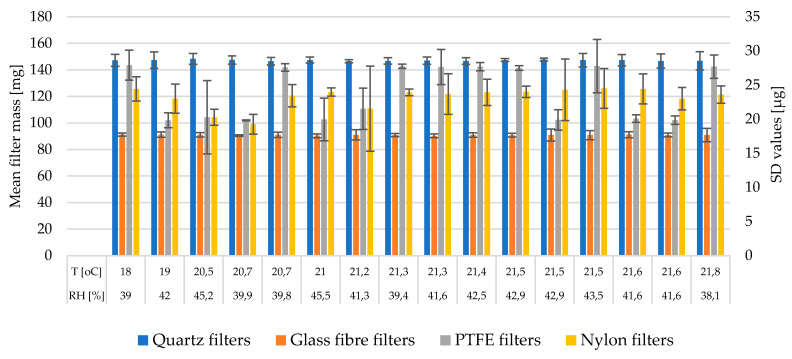
Mean filter mass (mg) and standard deviations (SD) (µg) of filters mass calculated as a weight difference between subsequent 24 h and 48 h weighing sessions under changing conditions of equilibration.

Figure 2 shows the average filter mass for each type of filter after 48h of conditioning (18–21.8 °C and 38.1–45.5% RH), where the x-axis informs about average conditions held in the weighing room in separate experiments. The typical masses measured were: 145–150 mg for Q filters, 90–91 mg for G filters, 140–143 mg and 99–103 mg for PTFE supported with a binder and plane one, respectively, and 115–130 mg for N filters. Mass spreads for individual filters were similar to each other regardless the mass of individual filters. A large variation in the filter mass was observed, with quartz and glass filters showing the best mass measurement repeatability between 24 and 48 h. The SD values of filter mass averaged within single filter type were Q (3.48 µg), G (2.18 µg), PTFE (8.85 µg), and N (10.84 µg); these were within the range of 40 µg set by the EN 14907:2005 standard [23], which means that there was an acceptable change in filter mass between subsequent weighing sessions. Weighing precision expressed as standard deviation of repeatability (or in other words the range min-max of the average SD values [µg] between weighing sessions, recorded in each batch of five unloaded filters) were Q (1.13–6.79 µg), G (0.57–5.09 µg), PTFE (0.35–27.58 µg), and N (2.47–32.08 µg). This means that the lowest precision between the first and second weighing sessions were observed for Teflon and nylon filters. This was probably more due to their susceptibility to electrostatic charges compared to quartz or glass filters, than due to their ability to absorb water from the air. 

Considering the data in Figure 2, greater differences in filter mass (SD values, µg) were found when the RH conditions were in the range of 42–45% RH than 39–41% RH; under the lower RH% range, the differences in filter mass between weighing sessions were Q (3.75), G (1.89), PTFE (6.37), and N (8.17); under the higher RH% range, the differences were in Q (3.28), G (2.27), PTFE (10.61), and N (12.70). This observation complies with data published by [12], indicating that more stable results in filter mass are obtained under 40% RH compared to those collected at moisture conditions closer to 50%. The effect of temperature on the variations in filter mass was also tested by dividing the dataset into two temperature ranges: masses measured at 18–21 °C and those measured at 21–22 °C. The differences in filter mass expressed as the averaged SD (µg) at 18–21 °C were Q (3.84), G (1.45), PTFE (10.64), and N (7.50); differences in filter mass at 21–22 °C were Q (3.27), G (2.62); PTFE (7.78), and N (12.85). It could be therefore concluded that in case of quartz and PTFE; higher temperature of conditioning provided greater stability in filter mass; unlike with nylon and glass one. This part of the experiment yields in consistent results, probably due to a narrow range of temperatures tested. The influence of temperature on the response of filter blanks should engage a larger batch of filters conditioned for a longer period of time—even few days—with a greater time resolution–weight measurement after each 24 h—under different temperatures—for example from 18–25 °C—and should include measurement of absolute variations in single filter mass from initial weighing. Longer observations could bring more consistent information on the influence of temperature on the deviation in filter mass. Observations from this study emphasize that temperature in these ranges affects filter mass rather ambiguously.

### 3.2. Effects of Humidity and Temperature on the Presence of Water in Filters Used Popularly for PM Collection

**Figure 3:**
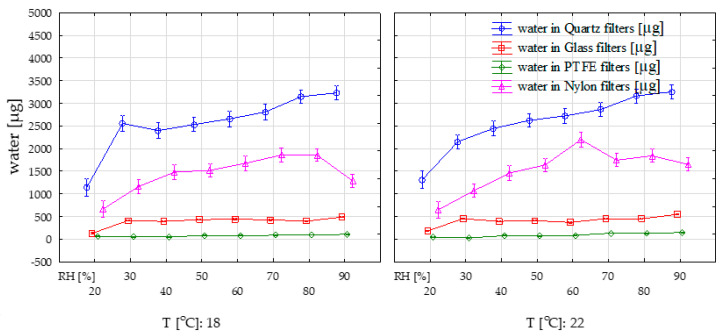
The interaction between temperature/humidity conditions and filter-bound water contents. RH (%)·Temperature (°C). Vertical bars represent the 0.95% confidence interval.

**Figure 4:**
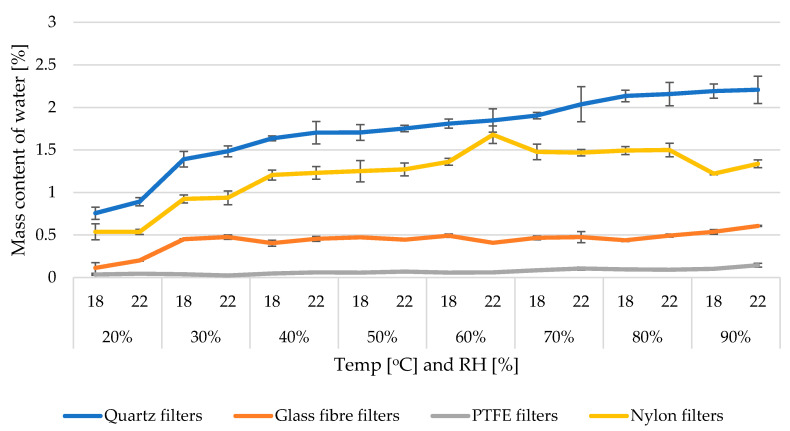
The contents of water in different types of filter blanks used popularly for the collection of PM samples calculated as a percentage of water mass (µg) to the filter mass (µg) under changing temperature and RH conditions.

Calculation of the amount of water in PM filters was started by testing the normality of filters weight under different temperature and RH% conditions using the Shapiro-Wilk test (*p* < 0.05). Due to a non-normal distribution of PTFE filters without binder (Figures S1 and S4), the median values were used as an estimator for the presentation of filter-bound water contents. All further comparisons regarding variations in filter mass/water contents under different temperature/humidity conditions were done using non-parametric statistical analyses. For simplification of the further discussion and easier visualization of the results, PTFE filters with and without binder were treated as one group (PTFE filters). Simple comparisons made on the dataset (Table 1) found that the median water contents in the whole tested humidity spectrum was 2727.8 µg for Q, 417.2 µg for G, 81.4 µg for PTFE, and 1582.7 for N filters. The ranges of water contents in filter blanks in the whole spectrum of tested temperatures and RH were 1013.7–3401.6 µg for Q, 111.7–556.2 µg for G, 20.8–188.2 µg for PTFE, and 509.1–2328.0 µg for N filters. The mass of water in terms of absolute values were therefore the highest in quartz fiber filters, followed by nylon, glass, and PTFE. Obtained results indicate that temperature and humidity simultaneously explain a statistically significant amount of variance in the “filter water contents” variable. However, humidity influences a greater extent of the variation in water contents (Figure 3). This is observable, especially under 18 °C for quartz filters, where a small rise in RH from 20 to 30% adds almost 1500 µg of water. The smallest average water content occurs in the case of filters made from PTFE. When recalculated per filter mass (Figure 4) these contents were 0.75–2.2% Q, 0.1–0.6% G, 0.02–0.14% PTFE, and 0.53–1.67% N. Both in terms of absolute mass and filter mass, the greatest hydrophilicity was shown by quartz filters. Variations in water contents under different temperature/humidity conditions were also greatest in quartz filters (Figure S2). This agrees with the results obtained by [16], who assessed the performance of quartz and Teflon filters for atmospheric water absorption when collecting PM.

Since those differences are probably due to different physical and chemical properties of filters, some exploratory quantitative analyses were performed to assess the effects of humidity and temperature on the variations of water absorption by filters. The most interesting observations were made based on Table 2, including two-factorial ANOVA calculations. To test the variance, we claim that both the temperature and humidity have a significant (*p* < 0.05) influence on filter water contents inside each group of filter types. The variance analysis breaks down variability into additive components whose number depends on the needs of the experiment. A comparison of individual resulting variances from a given factor and the error variance answers whether a given factor plays an important role in shaping the results of the experiment. By comparing (with the usage of the F test) the variance of the effect with the error variance (inside the tested group), we decide whether the group means of the effect under consideration differ significantly between themselves. If the division into groups takes place due to different levels of factors (in this example, temperature and humidity), we can detect a significant impact of the level of these factors on the effect (level) of the variable being tested–water contents inside filters. As presented in Table 2, the influence of temperature and humidity on the water contents in filter blanks were almost each time significant (*p* < 0.05). Both temperature and humidity differentiate the mean contents of the filter-bound water. The only exception occurs in quartz filters. When analyzing results presented in Table 2, it appears, that there is a reason to reject the null hypothesis about the interaction between temperature and humidity influence on the water contents in filters made from quartz (Q), due to negligible temperature effect. Probably the used temperature range was too narrow to be significant or this influence was overshadowed by the influence of the RH factor. The MS_Effect_ values were 97.91, 350.05, 128.570, and 50.24 times higher compared to MS_Residual_ values, for quartz, glass, PTFE and nylon filters, respectively, which determines high significance of RH [%] factor.

To check whether individual filters mass influence water sorption inside the weighing room we performed correlation analysis (Figure S3). The correlation between those variables were Q (r = 0.16), G (r = 0.04), PTFE (r = −0.24), and N (r = −0.017). This showed that filters mass or quartz filters and PTFE filters were slightly correlated with its tendency for water absorption. In the case of quartz filters, this correlation was positive, and in the case of PTFE, the correlation was negative. The scatter chart of the PTFE filters indicates two different masses since two types of those filters were used (Figure S1), but response to humidity and temperature conditions was similar in both types of PTFE material. While looking into the correlation between filter wettability (water capacity) and a rise in humidity conditions it was found that correlation coefficients were 0.86; 0.66; 0.83 and 0.61 for Q, G, N and PTFE filters respectively, thereby suggesting that quartz filters are most responsive to any changes in RH conditions. 

In order to compare the effect of water contents in filter blanks on the accuracy of filters weight measurements we compared a rise in absolute water contents (µg) measured between 40–50% RH with the differences in filter mass between subsequent weighing sessions under 40–50% RH. At 40% RH, the presence of water was Q (2398.7 ± 50.8 µg), G (391 ± 8.8 µg), N (1480 ± 41.5 µg), and PTFE (48 ± 0.7 µg) in the first experiment and in the replicate Q (2443.65 ± 276.82 µg), G (391.86 ± 14.62 µg), N (1453.84 ± 57.22 µg), and PTFE (75 ± 1.68 µg); at 50% RH the water content was Q (2529 ± 133.80 µg), G (429.78 ± 7.67 µg), N (1517.42 ± 256.81 µg), and PTFE (81.8 ± 2.37µg) in first experiment and in the replicate Q (2621.0 ± 112.9 µg), G (408.7 ± 16.2 µg), N (1639.0 ± 32.7 µg), and PTFE (69.8 ± 4.2 µg) (Figure 4). Therefore the overall differences in water mass under those conditions were–after averaging results from first and second experiment–Q (154.15 µg), G (27.81 µg), N (111.71 µg), and PTFE (14.3 µg), while the differences in absolute filters mass under those conditions were Q (647.05 µg), G (210.8 µg), N (440.21 µg), and PTFE (28.4 µg). This means that variations in water contents under 40–50% RH constitute only 23% (Q), 13.3% (G), 24.5% (N), and 50% (PTFE) of all variation, creating gravimetric uncertainty. Such a simple comparison gave us the answer to the question of which part of the mass determination uncertainty comes from water deviation stimulated by differentiating moisture conditions. Most water mass was added to filters under high humidity conditions. Above 50% RH, the percentage of water content to the whole water identified was on average 85% for quartz filters, 79% for glass filters, 53% for PTFE and 73% for nylon filters. This means that the rise of water sorption for most hydrophobic Teflon filters is stable between 20–50% RH and 50–90% RH.

## 4. Discussion

The only information given in a proper standard [23], regarding deviations in the mass of filter blanks under varying temperature/humidity conditions is that if differences between replicated gravimetric measurements of unsampled filters changed by less than 40 µg–in case of LVS–then the unloaded filters can be treated as condition one and further measurements can proceed. The contribution of gravimetric uncertainty due to humidity variations in the weighing room, however, given in the mentioned standard and included in the final uncertainty budged, does not inform which part of this uncertainty results from filter-bound water contents. The deviation in filter weight due to moisture conditions inside the weighing room should be further assessed using appropriate and sufficiently precise analytical methods. When comparing the amount of filter-bound water with sample blanks—understood as the water content in a closed volume of air under conditions corresponding to filter exposure conditions—measured in this study using Karl Fischer method–42.21 ± 13.3 µg with the levels of water contents typically found in quartz, glass, nylon, or highly hydrophobic Teflon filters–under 50% RH, 0.056–0.067% of filter mass, and in absolute terms 32–52.9 µg– the KF method, due to its detection limits and reproducibility is probably the best analytical solution for such determinations. The whole discussion concerning the quantitative assessment of water contents in unsampled (non-exposed) filters should start from the definition of quality assurance and quality control water determinations using chemical methods. When looking into literature data concerning filter-bound or PM-bound water contents it becomes obvious about how small analytical levels are we speaking about [13,16,24,25], and how many factors–apart from filter-bound water–plays role in falsifying filter weight. In 1997, the US Environmental Protection Agency (US EPA) established the Federal Reference Method [26] for collecting fine dust (PM2.5) on polytetrafluoroethylene (PTFE) filters. According to this method, the gravimetric measurement of PM mass consists of subtracting the mass of the empty (unexposed) filter from the mass of the filter containing the PM2.5 dust sample (filter after exposure). In order to minimize measurement errors resulting from the variability of environmental weighing conditions of filters, US EPA [27] has established quality assurance guidelines recommending 24-h conditioning of PTFE filters (both unloaded and loaded PM) in the temperature range 20–23 ± 2 °C and relative humidity (RH) 30–40% ± 5% RH. Protocols developed by the European Committee for Standardization (CEN) for measuring the mass of coarse particulates (PM_10_) [3] and fine (PM_2.5_) particles [23] allow greater diversity in the range of filter materials used to collect PM and provide longer conditioning time (48 h), as well as more stringent temperature conditions (20 ± 1 °C) and a wider RH range (50 ± 5%) compared to EPA guidelines [22]. Results obtained in this study indicate that 39–41 RH% conditions were most recommended for filters conditioning, due to small variations in filter mass between weighing sessions and in accordance with [22] findings. Few research studies also point that the mass of filter blanks varies to a greater extent when RH is close to 50%; while under lower RH those changes are smaller. For example in [12] the difference between average weighing sessions of blank filter mass after conditioning for 48 h and 72 h at 20 °C/50%RH; 18 °C/40%RH; 22 °C/60% RH and 20 °C/50%RH (repeat) was tested. The authors found that the lowest differences in filter mass occurred under 18 °C and 40% RH oscillating around 9 µg for quartz filters after 24 h sampling; −2 µg for glass filters without binder after 24 h sampling, 30 µg for glass filters with organic binder after 72 h sampling; 18 µg for glass filters with organic binder after 24 h and 49 µg for PTFE-bonded glass fiber filters after 24h sampling. Under higher humidity conditions 22 °C/60% RH those values were 99 µg; 125 µg; 120 µg; −67 µg and −19 µg, respectively. Similar conclusions regarding preferable usage of 40% RH during conditioning were obtained by [25], who performed buoyancy corrected gravimetric analysis of blank PTFE filters under increasing percent RH conditions while the temperature was held constant 21.5 ± 0.4 °C (n = 9462 readings). He found that the difference in PTFE filters mass ΔM (mean ± SD) µg under 15.9; 20.6; 30.6; 40.5; 49.9; 59.6 RH% were as follows (−0.9 ± 0.6); (0.7 ± 0.7); (0.4 ± 0.6); (−0.2 ± 0.5); (1.3 ± 0.4); (2.9 ± 1.5). The lowest variation in filter mass was therefore observed under 40.5 RH%. Results from our study indicate that the mass of filters, without any collected particulate matter varied with humidity and temperature depending not only on filter material but also between filters of nominally the same material, even within specified and rather narrow temp/RH% ranges. In this study filter-bound water contents variation per 10% change in RH was 200 µg(Q); 65 µg(G); 127 µg(N); 13 µg for PTFE with a binder, and 12 µg for PTFE filters without a binder (calculated based on regression results presented in Figure S4). In case of Teflon filters this would create a 12–13% relative errors in 100 µg net mass. This is in some contradiction with observations made by [12], who analyzed weight to humidity variation for blank filters and estimate that glass exhibited about 19 µg variations in filter weight per 10% change in RH, while in case of quartz fiber filters this change was approximately 95 µg per 10% change in RH. The levels of weight variation obtained by [12] are somewhat lower compared to ours; however, this testing was narrowed to only 40–60% RH%, while ours analyzes were extended from 20 to 90% RH, which fact can significantly influence the regression results. Even small variations in mass measurement may contribute significant errors to calculated concentrations. Hänninen et al. [28] used 23 laboratory blank Teflon filters, weighted 951 times, to statistically determine the factors mostly affecting the observed filter weight. He found that under 50% RH, air buoyancy variation and filter storage time are an order of magnitude more important factors than humidity in the weighing-room. Relative humidity was found to have a statistically significant (*p* = 0.005) but very weak (R2 = 0.8%, k3 = 0.0546 µg/RH%) effect on blank filters. Of course, variations in gravimetric measurements cannot be compared with the variations in filter-bound water, since gravimetric uncertainty budged result not only from the effects of humidity but several other factors included in a standard procedure. The EN Standard [23] tries to deal with the problem of mass uncertainty during gravimetric determinations by setting stable temperature and humidity conditions to 20 °C ± 1 °C and 50% ± 5% RH respectively. It must be however remembered that the direction from which this optimal (50% RH) condition is approached plays an important role in its final mass result. If 50% RH is approached from a lower RH, the filter blank will contain less water at the equilibrium compared with situation when 50% RH is approached from higher RH. This phenomenon is known as hysteresis effect [29] and well described in the literature. Defining univocally the optimal time and starting conditions of preconditioning is therefore a hard task and will depend on filter type. Few previously published works regarding filters conditioning tried to assess the shape of filters hysteresis loop by carrying initial weighing under stabilized temp and humidity conditions, subsequently exposed to higher/lower temp and humidity conditions. Such raise/reduction in temp and humidity is usually reflected only in the measurements of the change in filter mass by measuring differences in their mass between subsequent weighing sessions (performed on a single batch of filters). Nothing is however known regarding the influence of those temp/RH% variations in absolute filter water contents. Some authors indicate that longer filter storage influences greater losses of volatile compounds (e.g., nitrates; some organic compounds and water) [30,31]; and therefore, mass loss. Somewhat different results were obtained by Hänninen et al. [28] who observed small mass gain under after 30 days of storage (in total 4.308 µg) compared to 10-day long period (in total 2.896 µg). Some alternative method to control humidity and temperature effects could be the use of weighing batch blanks. Such approach will unfortunately greatly increase the number of weighing and handling to be performed and the fact that each net mass result would be built using four, instead of two, raw mass observations.

This work is a significant contribution to the existing database on filter conditioning process, as it gives a clear answer to the question: which portion of the mass deviation originates from water content variations. The second advantage of this work compared to the quantification of the effect of humidity on the mass of blank filters based on their weight measurements is that KF titration method measures only water, while simple gravimetric-based comparisons account for other compounds influencing weight uncertainty, such as the loss of volatile compounds. The proposed method is not free from limitations, among which the most important one is determination of total (possibly desorbed) water without its speciation into free-absorption water and water strongly bound to filter material. Future analysis should be directed not only to quantitative but also qualitative measurement of filter-bound water contents through the RAMP analysis water contribution where different classes of water are desorbed under different temperatures.

Existing guidelines and regulations define the procedures to ensure accurate, reproducible filter weighing and specify which filters should be used and which weighing system to use for the PM measurement. Currently there are no recommendations for filter-bound or PM-bound water determinations. For PM-bound water assessments, the usage of a particular filter type will depend on other analysis purposes, since one PM sample (one filter) is usually used for multiple analytical purposes. Quartz fiber filters, characterized by reasonable collection efficiency and low cost are the group of filters most increasingly used in air sampling; however containing a large amount of Al and Si and therefore not recommended for elemental analysis, similarly as in case of water determinations. PTFE or PTFE-polypropylene backed filters, while advised for water analysis are typified with low melting point, approximately 60 °C and therefore inappropriate for qualitative determinations of PM-bound water research, because water bound to carbonaceous material requires evaporation at temperatures around 400 °C (pyrolysis). Another problem appearing during water determination from PTFE filters in the presence of organic binder or polypropylene matrix, which can interfere with subsequent analysis (for example qualitative assessment of strongly-bound water), so the filter must be flash-fired to remove the binder material. Such action can cause a loss in tensile strength and filter damage [6,32]. This work does not include the whole spectrum of analytical problems arising during filter-bound water determinations, however, gives some certain view on possible analytical problems encountered.

## 5. Conclusions

The present work assesses the effects of the alteration of humidity and temperature on the weight of filter blanks made from different materials and analyzes the relations between variations in temp and RH% on the amount of water sorption by those filters. This work assessed the alteration of humidity and temperature in a balance room attributing the deviation in the measurement of the mass of the filter. It also provides the quantitative determination of filter-bound water contents rarely found in the literature. The results indicate that the alteration of humidity and (or) temperature in a balance room attributes to the deviation of the measurement of filters mass and thus can affect the gravimetric measurements of ambient particulate matter. It was found that quartz and nylon filters are most susceptible to changes in water content under changing air humidity, and therefore less suitable for determining the mass of fine and ultra-fine particles. The best filter media exhibiting lowest water contents under variable humidity conditions are PTFE filters, followed by glass filters.

It was also found that regardless of filter type, the average weight differences of unloaded filters under 21–22 °C range (*p* < 0.05) degrees were significantly higher compared to those measured under 18–21 °C. A similar relationship was observed in case of fluctuations in relative humidity—the average differences in filter masses at 39–41% RH were lower than under 42–45% RH humidity conditions. Obtained results confirm the observations made by EPA (Quality Assurance Guidance Document 2.12: Monitoring PM_2.5_ in Ambient Air Using Designated Reference Class 1 Equivalent Methods; [27], suggesting that filter conditioning ensuring minimal mass fluctuations should oscillate within: 20–23 °C; 30–40% RH ± 5%. This means that filter conditioning (especially in terms of air humidity) proposed in the standard (EN 12341; [3]) and amounted to 20 ± 1 °C; 50% ± 5% should be reduced to levels recommended by US EPA [27]. The results presented here indicate that for PM-bound water quantification the best choice are PTFE filters. When taking into account chemical analyzes of those by Karl Fischer method the maximum evaporation temperature cannot exceed 60 °C, which at the same time significantly extends the analysis time, e.g., compared to glass filters. This aspect is important especially in measurement campaigns with many samples (for example by using multiple-stages cascade impactors). Apart from this limitation PTFE filters are the best choice when assessing PM-bound water contribution. Further investigations should be directed on typing different classes of water inside filters for PM collection and on intermolecular interactions between the different filter media and water molecules using the Karl Fischer method. This work is an important contribution to the state of the art in the field of assessing the suitability of various types of filter materials for specific chemical analyzes and monitoring campaigns.

## Figures and Tables

**Table 1:** Descriptive statistics of the water contents (µg) in filter blanks.

Variable	N	Mean	SD	Median	Min	Max	Lower Quartile	Upper Quartile	Perc. 25%	Perc. 75%
Water in Q	72	2608.6	588.2	2727.8	1013.7	3401.6	2389.4	3045.5	2389.4	3045.5
Water in G	78	401.2	99.1	417.2	111.7	556.2	392.4	449.0	392.4	449.0
Water in PTFE	79	82.8	33.6	81.4	20.8	188.2	53.8	102.8	53.8	102.8
Water in N	80	1474.8	435.0	1582.7	509.1	2328.0	1129.9	1824.3	1129.9	1824.3

**Table 2:** Results from single-variate analysis of variance (2-way factorial ANOVA).

**Water in Quartz Filters [µg]**					
**Effect**	**df**	**SS**	**MS**	**F**	***p***
Intercept	1	445,699,653.0	445,699,653.0	1,543,273.0	0.0000
RH [%]	7	19,793,321.0	2,827,617.0	97.91	0.0000
T [°C]	1	931.0	931.0	0.03	0.8581
RH [%] · T [°C]	7	441,994.0	6,142.0	2.19	0.0498
Residual	54	1,559,529.0	28,880.0		
Total	69	21,937,020.0			
**Water in Glass Filters [µg]**					
**Effect**	**df**	**SS**	**MS**	**F**	***p***
Intercept	1	10,737,087.0	10,737,087.0	50,201.18	0.0000
RH [%]	7	524,077.0	74,868.0	350.05	0.0000
T [°C]	1	5,363.0	5,363.0	25.08	0.0000
RH [%] · T [°C]	7	30,828.0	4,404.0	20.59	0.0000
Residual	54	11,550.0	214.0		
Total	69	574,048.0			
**Water in PTFE Filters [µg]**					
**Effect**	**df**	**SS**	**MS**	**F**	***p***
Intercept	462,169.5	462,169.5	6,992.602	0.0000	
RH [%]	7	59,483.8	8,497.7	128.570	0.0000
T [°C]	1	2,369.9	2,369.9	35.857	0.0000
RH [%] · T [°C]	7	13,359.3	1,908.5	28.875	0.0000
Residual	54	3,569.1	66.1		
Total	69	82,404.7			
**Water in Nylon Filters [µg]**					
**Effect**	**df**	**SS**	**MS**	**F**	***p***
Intercept	1	149,486,001.0	149,486,001.0	5,759.898	0.0000
RH [%]	7	9,196,900.0	1,313,843.0	50.624	0.0000
T [°C]	1	147,942.0	147,942.0	5.700	0.0204
RH [%] · T [°C]	7	788,726.0	112,675.0	4.342	0.0007
Residual	54	1,401,456.0	25,953.0		
Total	69	11,582,623.0			

Note: SS—sum-of-squares (SS), df (degrees of freedom)—the total number of values minus 1, MS—mean square value computed by dividing a sum-of-squares value by the corresponding degrees of freedom; F ratio is computed by dividing the MS value by another MS value (in case of ANOVA without repeated measures)—MS value is the MS residual.

## Supplementary Materials

The following are available online at https://www.mdpi.com/1660-4601/17/14/5180/s1, Figure S1: Results from normality testing using Shapiro-Wilk test (*p* < 0.05). Distribution of filter masses [µg] under differentiating humidity and temperature conditions, Figure S2: Median, 25 and 75% percentiles and min/max values of water contents (calculated as absolute mass [µg]) in different types of filter blanks under variable humidity conditions (20–90%), Figure S3: Scatter charts presenting the strength of the correlation between averaged filter mass [mg] and absolute water contents [µg], Figure S4: Scatter charts presenting the regression results between averaged filter-bound water contents [µg] and humidity [%], together with correlation coefficients (r).
