# Supplementary material for: Water Sorption by Different Types of Filter Media Used for Particulate Matter Collection Under Varying Temperature and Humidity Conditions
Source: Int J Environ Res Public Health. 2020 Jul 17;17(14):5180. doi: 10.3390/ijerph17145180 (PMC7400541; doi:10.3390/ijerph17145180)
Supplement: Supplementary file 1 [file ijerph-17-05180-s001.pdf]

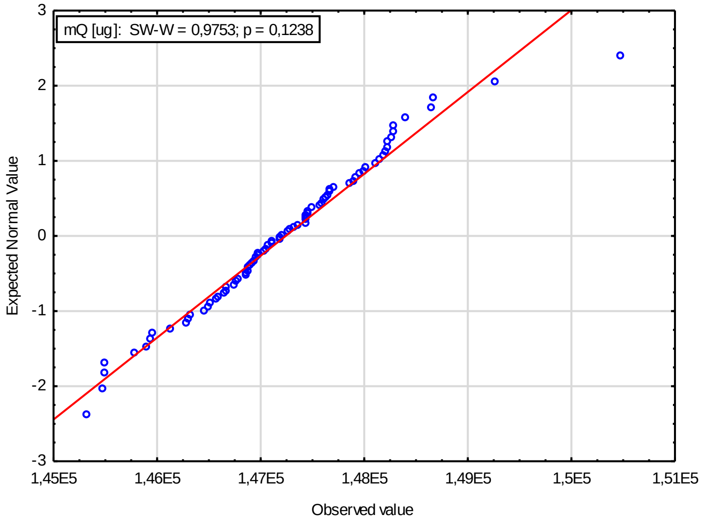
mQ [ug]: SW-W = 0,9753; p = 0,1238
Expected Normal Value
Observed value
3
2
1
0
-1
-2
-3
1,45E5
1,46E5
1,47E5
1,48E5
1,49E5
1,5E5
1,51E5

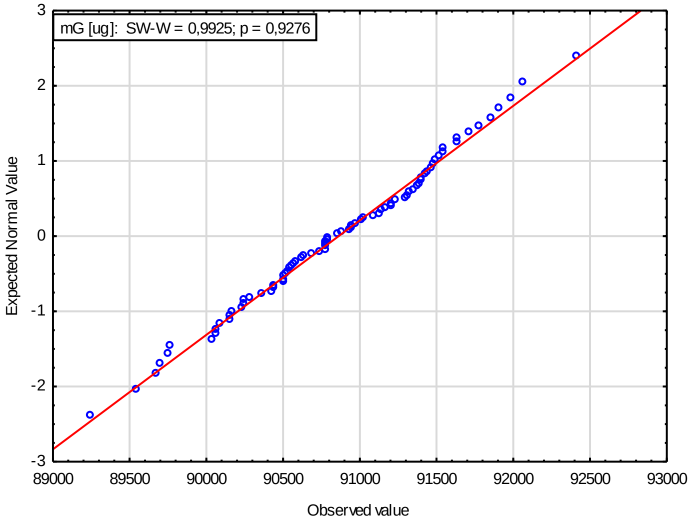
mG [ug]: SW-W = 0,9925; p = 0,9276
Expected Normal Value
Observed value
3
2
1
0
-1
-2
-3
89000
89500
90000
90500
91000
91500
92000
92500
93000

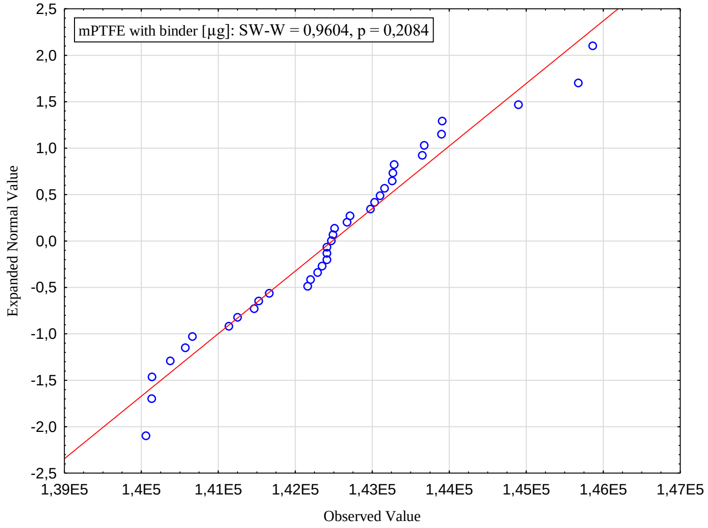
mPTFE with binder [µg]: SW-W = 0,9604, p = 0,2084
Expanded Normal Value
Observed Value
2,5
2,0
1,5
1,0
0,5
0,0
-0,5
-1,0
-1,5
-2,0
-2,5
1,39E5
1,4E5
1,41E5
1,42E5
1,43E5
1,44E5
1,45E5
1,46E5
1,47E5

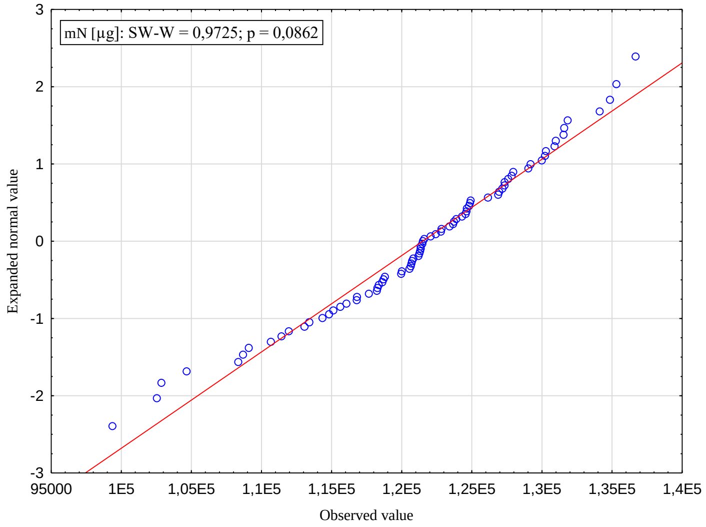
mN [µg]: SW-W = 0,9725; p = 0,0862
Expanded normal value
Observed value
3
2
1
0
-1
-2
-3
95000
1E5
1,05E5
1,1E5
1,15E5
1,2E5
1,25E5
1,3E5
1,35E5
1,4E5

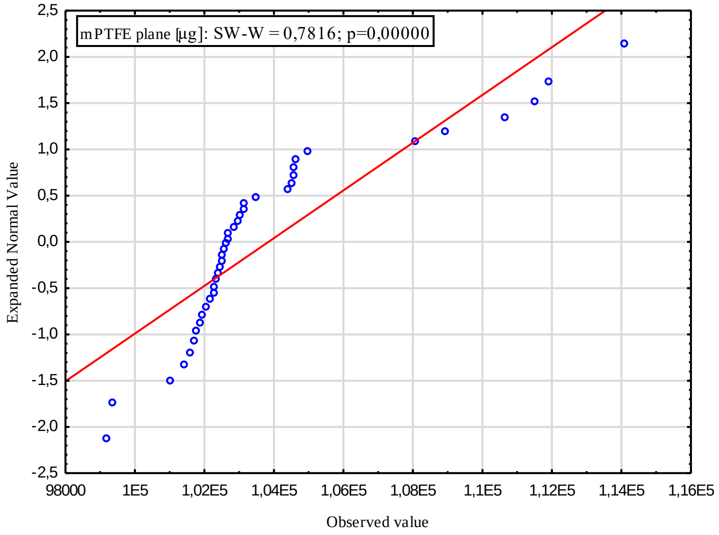

Fig S1. Results from normality testing using Shapiro-Wilk test ($p<0.05$). Distribution of filter masses [µg] under differentiating humidity and temperature conditions.

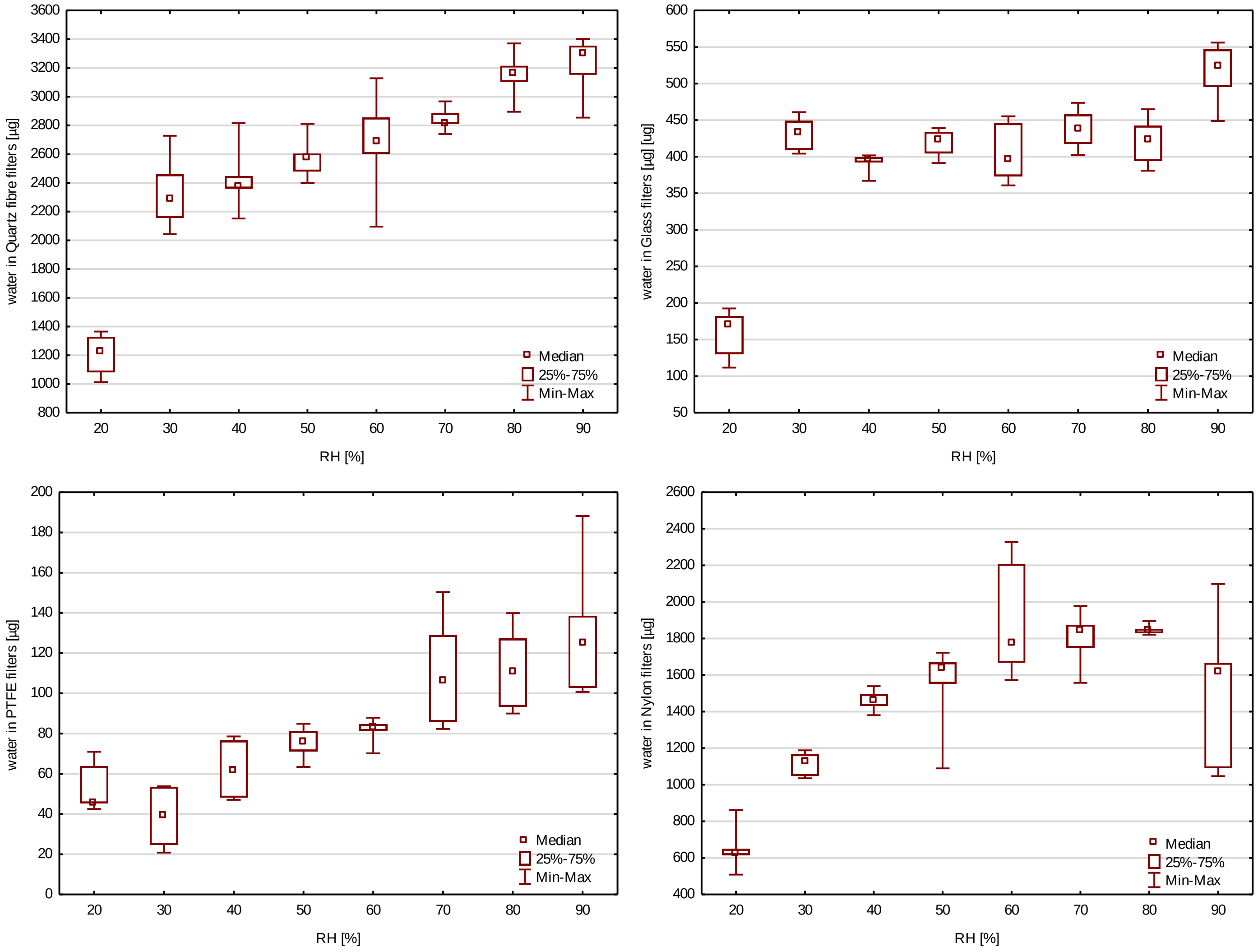

Fig S2. Median, 25 and 75% percentiles and min/max values of water contents (calculated as absolute mass [µg]) in different types of filter blanks under variable humidity conditions (20-90%).

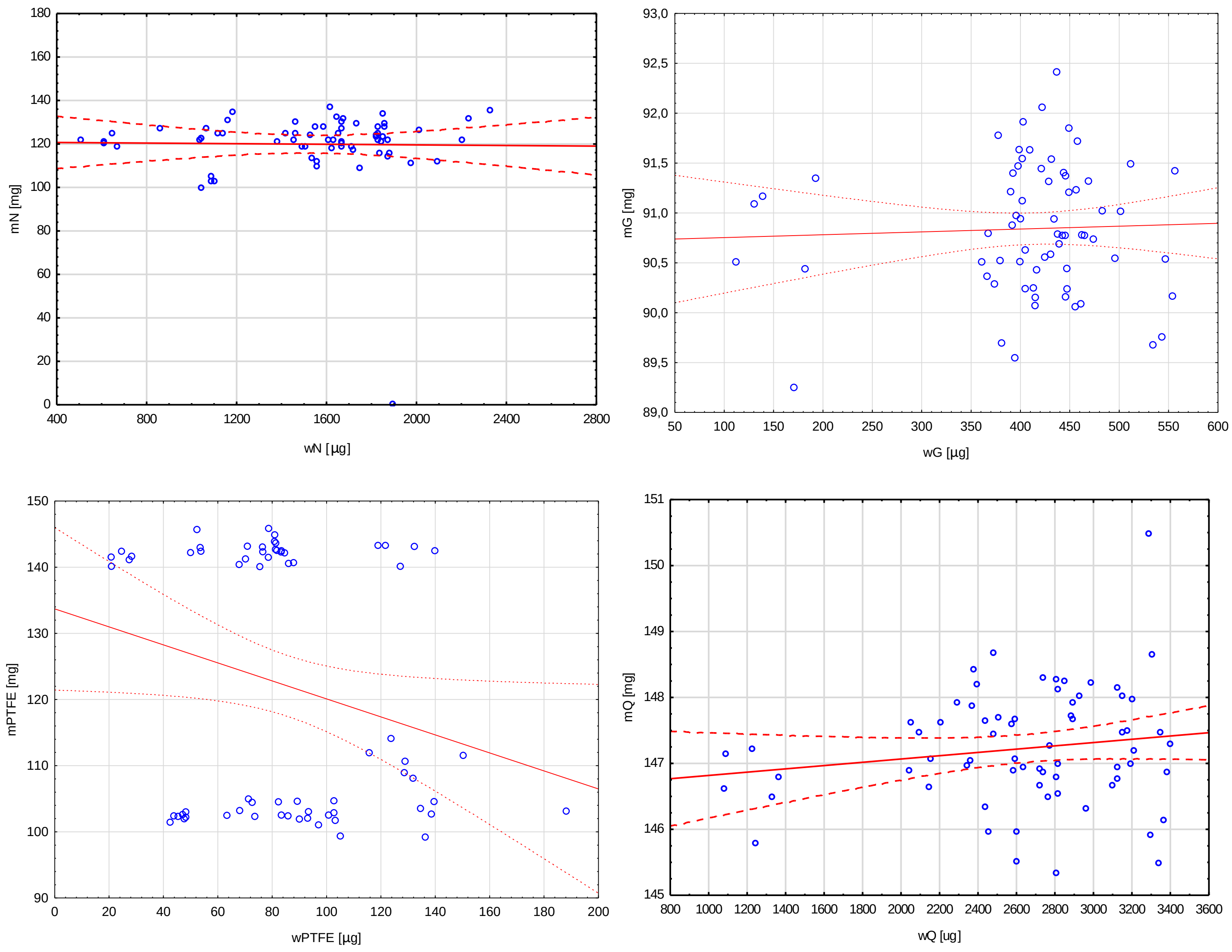

Fig S3. Scatter charts presenting the strength of the correlation between averaged filter mass [mg] and absolute water contents [µg]

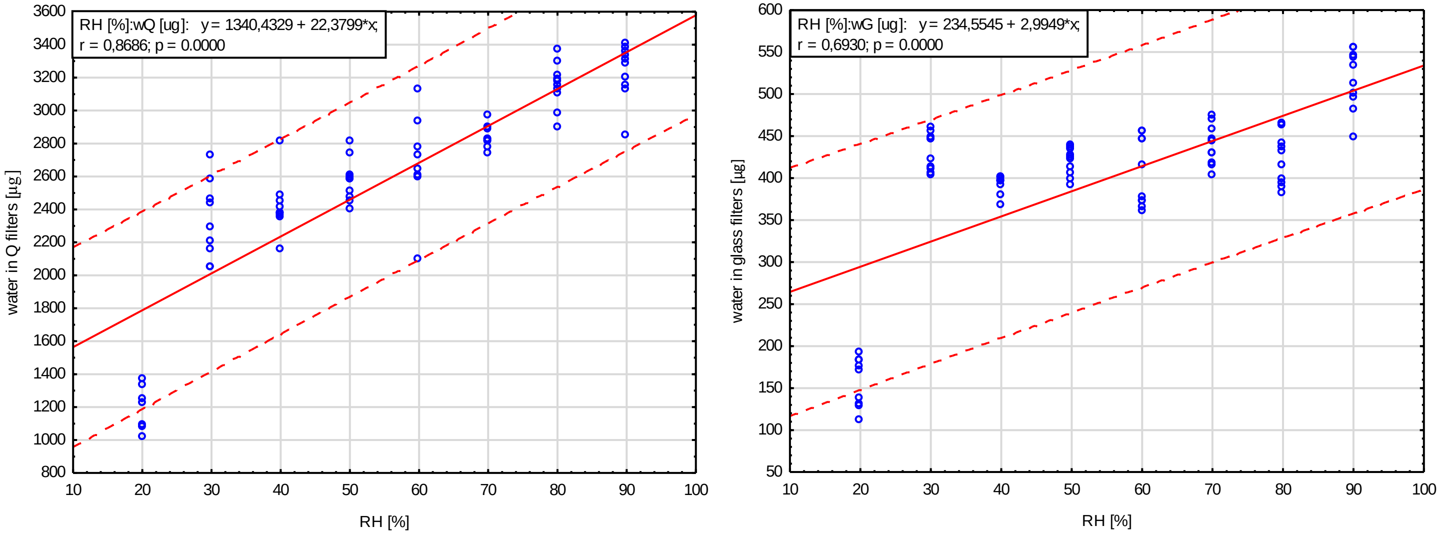
RH [%]:wQ [ug]: y = 1340,4329 + 22,3799*x;
r = 0,8686; p = 0.0000
water in Q filters [µg]
RH [%]
RH [%]:wG [ug]: y = 234,5545 + 2,9949*x;
r = 0,6930; p = 0.0000
water in glass filters [µg]
RH [%]

5

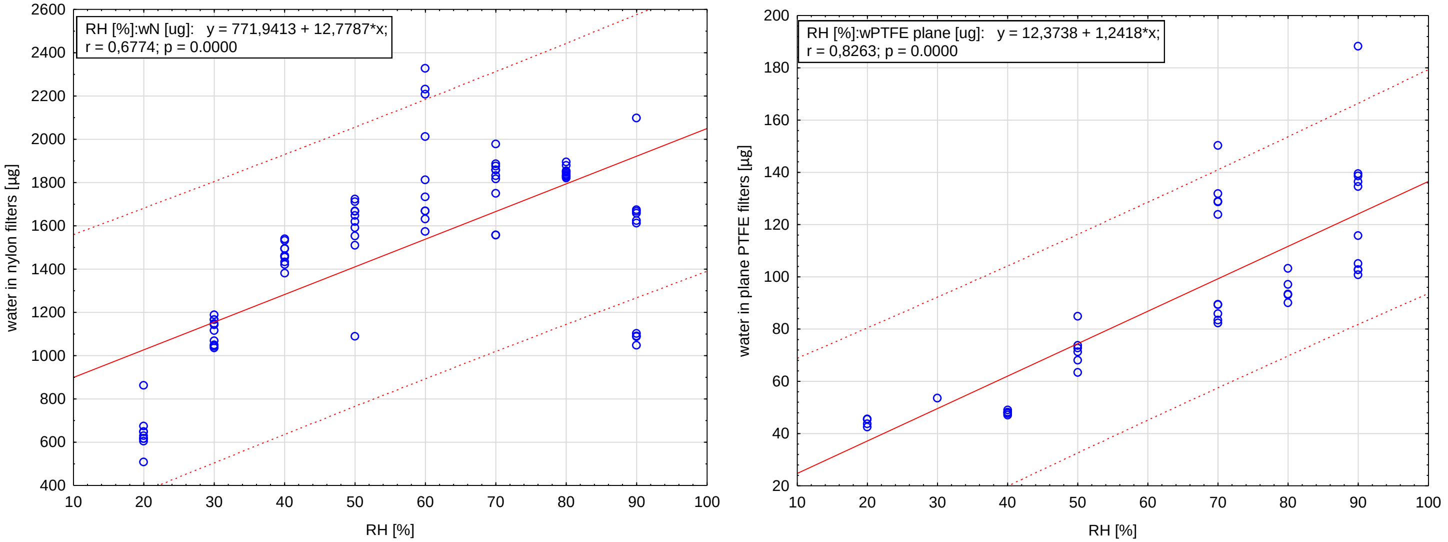
RH [%]:wN [ug]: y = 771,9413 + 12,7787*x;
r = 0,6774; p = 0.0000
water in nylon filters [µg]
RH [%]
RH [%]:wPTFE plane [ug]: y = 12,3738 + 1,2418*x;
r = 0,8263; p = 0.0000
water in plane PTFE filters [µg]
RH [%]

6

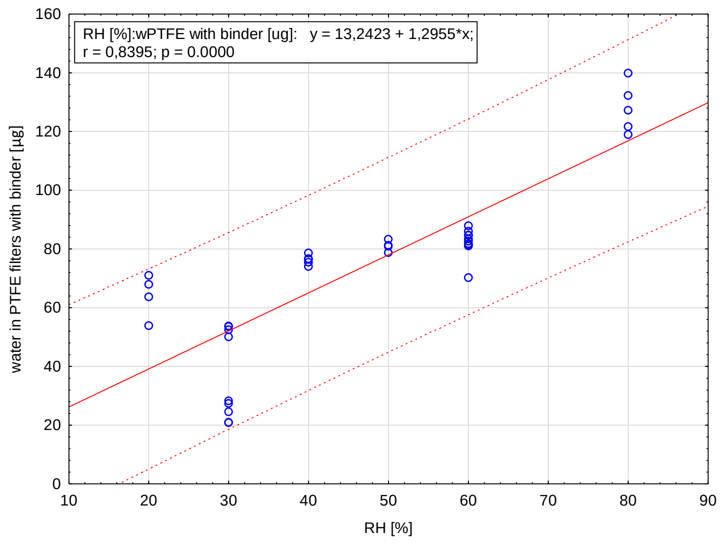

Fig S4. Scatter charts presenting the regression results between averaged filter-bound water contents [µg] and humidity [%], together with correlation coefficients (r).
